# Seroconversion to mRNA SARS-CoV-2 Vaccines in Hematologic Patients

**DOI:** 10.3389/fimmu.2022.852158

**Published:** 2022-05-12

**Authors:** Bruno Fattizzo, Marta Bortolotti, Nicolò Rampi, Francesca Cavallaro, Juri Alessandro Giannotta, Cristina Bucelli, Ramona Cassin, Matteo Claudio Da Vià, Giulia Galassi, Alessandro Noto, Loredana Pettine, Francesca Gaia Rossi, Mariarita Sciumè, Ferruccio Ceriotti, Dario Consonni, Wilma Barcellini, Luca Baldini

**Affiliations:** ^1^Hematology Unit, Fondazione IRCCS Ca’ Granda Ospedale Maggiore Policlinico, Milan, Italy; ^2^Department of Oncology and Oncohematology, University of Milan, Milan, Italy; ^3^Central Laboratory Unit, Fondazione IRCCS Ca’ Granda Ospedale Maggiore Policlinico, Milan, Italy; ^4^Epidemiology Unit, Fondazione IRCCS Ca’ Granda Ospedale Maggiore Policlinico, Milan, Italy

**Keywords:** myeloproliferative disorders, lymphoma, chronic lymphocytic leukemia, acute leukemia, multiple myeloma, COVID-19, SARS-CoV-2 vaccine

## Abstract

Hematologic patients show lower responses to SARS-CoV-2 vaccines, but predictors of seroconversion are lacking. In this prospective cohort study, hematologic patients undergoing SARS-CoV-2 mRNA vaccination at a single center in Milan, Italy, were sampled for anti-Spike and anti-Nucleocapsid IgG titer at 5 ± 1 weeks and at 3 months from the second vaccine dose. Patients (*N* = 393) received either BNT162b2 (Pfizer-BioNTech, 48%) or MRNA-1273 (Moderna, 52%), and 284 (72%) seroconverted and 100% persisted at 3 months. Non-response was higher in chronic lymphocytic leukemia (CLL) and lymphoma patients, and in those treated with small molecules and monoclonal antibodies. In myeloid neoplasms, lower responses were detected in patients with acute myeloid leukemia treated with venetoclax plus hypomethylating agents and in patients with myelofibrosis receiving ruxolitinib. Multivariable analysis showed that seroconversion was favorably associated with a diagnosis other than indolent lymphoma/CLL [OR 8.5 (95% CI 4.1–17.6)], lack of B-cell-depleting therapy [OR 3.15 (1.7–5.9)], and IgG levels within the normal range [OR 2.2 (1.2–4.2)]. We developed a simple algorithm according to these 3 risk factors [(A) diagnosis of indolent lymphoma/CLL, (B) B-cell-depleting treatment, and (C) low IgG] to predict non-response. IgG levels and treatment may be modifiable risk factors and should be considered for timing of vaccine administration.

## Highlights

1. Lower seroconversion was predicted by the presence of lymphoproliferative disease, B-cell-depleting treatment, or hypogammaglobulinemia.

2. Allowing time from monoclonal antibody infusion, holding on with small molecules, and waiting for IgG recovery may optimize seroconversion.

## Introduction

There is increasing concern about the efficacy of SARS-CoV-2 vaccines in fragile patients, including hematologic ones that have been recently reported to respond less to mRNA vaccines (1–3). While some reports described the association between non-response and diagnosis of indolent non-Hodgkin lymphomas (NHLs), both therapy-naïve and those treated with B-cell-depleting therapies (4, 5), data are less clear for myeloid diseases and patients with non-oncological hematologic conditions (6). Furthermore, no routine laboratory predictors of seroconversion after SARS-CoV-2 vaccine have been reported. In this study, we prospectively evaluated the rate of seroconversion after mRNA SARS-CoV-2 vaccines in hematologic patients undergoing vaccination at a single center in Milan, Italy. Patients were selected according to frailty criteria, including those on recent/ongoing treatment with potentially immunosuppressive drugs, stem cell-transplanted patients, or those who are candidates for immunosuppressive therapy. Anti-Spike persistence was also evaluated after 3 months from the last vaccine dose. We focused on clinical and laboratory risk factors for lower seroconversion, both non-modifiable and modifiable, and developed a simple algorithm to aid the clinician in choosing the timing of vaccination.

## Methods

### Study Design and Patient Selection

In this study, we prospectively evaluated hematologic patients undergoing SARS-CoV-2 mRNA vaccination from March until June 2021 at a single center in Milan, Italy. Patients were selected and prioritized for vaccination as per indications of the Italian Ministry of Health according to the following criteria: ongoing treatment with potentially immunosuppressive drug, recent chemo-immunotherapy (<6 months), stem cell transplant (SCT) within the last 12 months, and indication to receive immunosuppressive therapy in the next month (7). The study was conducted in accordance with the Declaration of Helsinki, and patients gave informed consent.

### Experimental Procedures

Patients were sampled and tested for anti-Spike and anti-Nucleocapsid IgG titer at 5 + 1 weeks from the second vaccine dose. Subsequently, positive cases were re-tested at 3 months from the last vaccine dose to assess the persistence of anti-Spike antibodies and/or the development of anti-Nucleocapsid antibodies as a sign of breakthrough COVID-19 infection.

To evaluate seroconversion, the tests Elecsys^®^ Anti-SARS-CoV-2 S and Elecsys^®^ Anti-SARS-CoV-2 were used [Elecsys^®^ Anti-SARS-CoV-2 S. Package Insert 2020-09, V1.0; Material Numbers 09289267190 and 09289275190]. These are immunoassays that use a recombinant protein representing the receptor binding domain (RBD) of the SARS-CoV-2 spike protein (S) or the nucleocapsid (N) antigen in a double-antigen sandwich assay format, which favors detection of high-affinity antibodies against SARS-CoV-2 S or N. The tests detect antibody titers, which have been shown to positively correlate with neutralizing antibodies in neutralization assays (8, 9). The positive cutoff of the test is a titer of 0.8 U/ml, and titers may then be converted 1:1 into binding antibody unit (BAU/ml) as per recently published WHO guidelines (10, 11).

### Statistical Analysis

For statistical analysis, we used Wilcoxon rank-sum and chi-squared test to compare categorical and quantitative variables, respectively. We calculated seroconversion proportions and 95% confidence intervals (CIs) using the Agresti–Coull formula. Odds ratios (OR) of seroconversion were calculated by a multivariable logistic regression model containing variables of *a priori* clinical interest. The area under the curve (AUC) from a receiver operating characteristic (ROC) curve was then calculated. Based on logistic model results, we calculated the predicted probability of vaccine response and developed a simple algorithm according to these 3 risk factors [(A) diagnosis of indolent lymphoma/CLL, (B) B-cell-depleting treatment, and (C) low IgG]. To analyze anti-Spike levels at 1 and 3 months, we used univariate and linear regression on log-transformed variables. Statistical analyses were performed with Stata 17 (StataCorp. 2021).

## Results

### Baseline Features

We enrolled 393 patients (male/female ratio, 1.07; median age, 68 years; range, 18–95) with the following diagnoses: 43 acute leukemias (31 myeloblastic, AML, and 12 lymphoblastic, ALL), 80 lymphomas (11 HL, 35 indolent NHL, and 34 aggressive NHL), 28 chronic lymphocytic leukemia (CLL), 13 myelodysplastic syndromes (MDSs), 103 myeloproliferative neoplasms (MPNs; 34 chronic myeloid leukemia, 24 myelofibrosis, 23 polycythemia rubra vera, 6 essential thrombocythemia, and 14 unclassifiable or MDS/MPN), 110 plasma cell dyscrasias (PCD) (107 multiple myeloma and 3 amyloidosis), and 16 non-oncological hematologic conditions (5 autoimmune cytopenias, 7 paroxysmal nocturnal hemoglobinuria, and 3 cold agglutinin diseases). Overall, 78% of patients were on active treatment, 9% had received recent chemo-immunotherapy, 6% SCT within the last 12 months, and 7% were candidates to receive therapy in the next month. Regarding disease status, 71% of subjects were in response on therapy, 13% subjects were in response off treatment, 8% in non-response, and 7% had active disease. Patients received either BNT162b2 (Pfizer-BioNTech, 48%) or MRNA-1273 (Moderna, 52%) vaccine, and 284 (72%) mounted an IgG anti-Spike titer >0.8 U/ml (the cutoff of our laboratory, converted 1:1 to BAU/ml). As shown in **Table 1**, responsive patients were younger than non-responders (*p* = 0.04) and displayed higher levels of lymphocytes (*p* = 0.001) and neutrophils (*p* = 0.04; patients with lymphocytes >5 × 10^9^/L and/or neutrophils >6.5 × 10^9^/L were excluded). Moreover, patients that seroconverted displayed greater values of IgG (*p* = 0.002) and IgA (*p* = 0.03); this was confirmed even after removing PCD from the analysis (*p* < 0.0001 for both).

**Table 1:** Laboratory features of patients divided according to positivity or negativity of anti-Spike IgG antibodies.

	Negative (*n* = 109)	Positive (*n* = 284)	*p*
Age, years	69 (20–89)	67 (18–95)	0.04
IgG mg/dl, all patients	615 (45–4,643)	889 (111–4,189)	0.002
IgG mg/dl, excluded PCD	655 (211–2,015)	995 (132–2,132)	0.0001
IgA mg/dl, all patients	68 (3–323)	98 (0–4,446)	0.03
IgA mg/dl, excluded PCD	77 (3–323)	119 (0–502)	0.0001
Lymphocytes ×10^9^/L	0.97 (0.22–4.8)	1.47 (0.28–4.5)	0.001
Neutrophils ×10^9^/L	2.5 (0.08–6.46)	2.73 (0–33–6.2)	0.04

PCD, plasma cell dyscrasia.

Values are given as median (range).

### Predictors of Seroconversion

**Figure 1:**
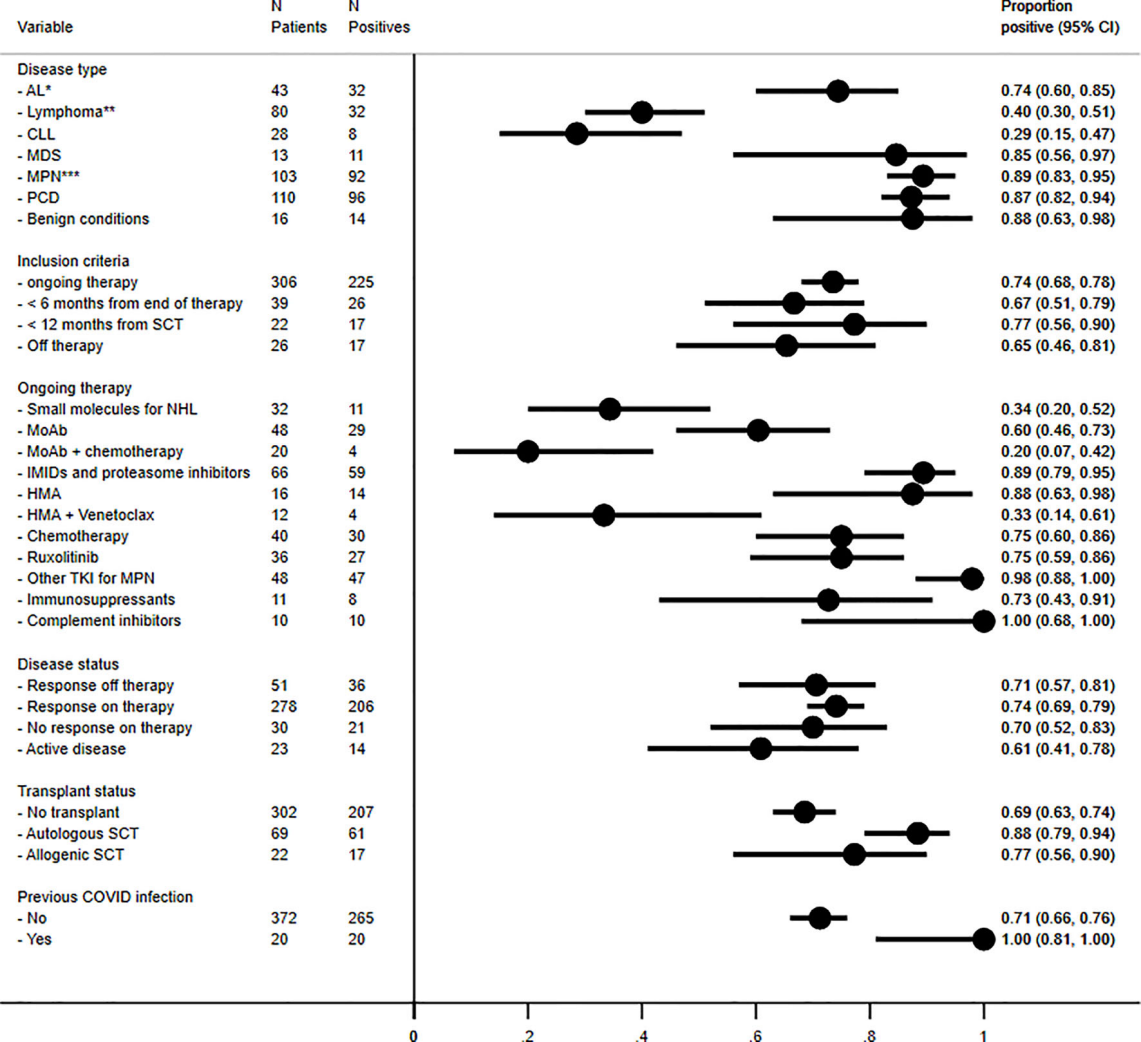
Frequency of seroconversion for anti-Spike antibodies according to disease type, inclusion criteria for vaccination, ongoing treatment, disease status, transplant, and previous COVID-19 infection. In details, *acute myeloid leukemia, *N* = 31, seroconverted in 71% of cases; acute lymphoblastic leukemia, *N* = 12, in 83%. **Hodgkin lymphomas, *N* = 11, seroconverted in 80% of cases; aggressive non-Hodgkin lymphomas, *N* = 34, in 48% of cases; and indolent NHL, *N* = 35, in 20% of cases. *** Chronic myeloid leukemias, *N* = 36, seroconverted in 100% of cases; primary myelofibrosis, *N* = 24, in 67% of cases; polycythemia vera, *N* = 23, in 91%; essential thrombocythemia, *N* = 6, in 100%; and unclassifiable myeloproliferative neoplasms, *N* = 14, in 100%. AL, acute leukemia, CLL, chronic lymphocytic leukemia, IMIDs, immunomodulating agents, MDS, myelodysplastic syndrome, MoAb, monoclonal antibodies, MPN, myeloproliferative neoplasms, PCD, plasma cell dyscrasias, TKI, tyrosine kinase inhibitors.

**Figure 1** shows the frequency of seroconversion according to clinical characteristics. Regarding hematologic diagnosis, the highest proportion of non-responders was observed in CLL and lymphoma patients (71% and 60%, respectively) as compared to <30% for other diseases (*p* < 0.001) (**Figure 1**).

#### - Lymphoid and Plasma Cell Diseases and Their Treatments

Among lymphomas, indolent NHL displayed the lowest frequency of seroconversion (20%), followed by aggressive NHL (48%), while HL seroconverted in 80% of cases. Treatment status had a significant impact (*p* < 0.001), with a lower rate of seroconversion (<50%) in patients on active therapy with small molecules for NHL and CLL (ibrutinib, idelalisib, and venetoclax, *p* < 0.001) and monoclonal antibodies (MoAbs; rituximab, obinutuzumab, brentuximab, daratumumab, elotuzumab, and belantamab) alone (*p* = 0.04) or in combination with chemotherapy (*p* < 0.0001). Finally, ALL patients seroconverted in 83% of cases.

#### *-* Myeloid Neoplasms and Their Treatments

Regarding myeloid neoplasms, seroconversion was observed in about 70% of cases, clearly better than in lymphoid diseases. However, lower responses were detected in particular settings including AML treated with venetoclax in combination with hypomethylating agents (HMA, 33% versus 88% in HMA alone, *p* < 0.01) and in primary myelofibrosis (67% versus >90% in other MPNs, *p* = 0.001) and in those treated with ruxolitinib (75% versus 98% with other TKIs, *p* = 0.001).

#### *-* Benign Conditions and Their Treatments

Regarding patients with a benign condition, 14/16 (88%) mounted anti-Spike IgG response to vaccine. This group included 10 patients on complement inhibitors: 7 paroxysmal nocturnal hemoglobinuria on C5 inhibitor eculizumab/ravulizumab (*N* = 6) or on oral factor B inhibitor (*N* = 1), and 3 cold agglutinin disease on C1s inhibitor sutimlimab. The 2 non-responders were patients with Evans syndrome (association of autoimmune hemolytic anemia and/or immune thrombocytopenia and/or neutropenia) who were on active treatment with immunosuppressive agents (steroids and rituximab).

#### *-* Other Predictors of Seroconversion

Regarding disease status, no important variation of seroconversion proportions was observed (*p* = 0.55), but in all disease groups (lymphoid, myeloid, and benign forms), patients in response off therapy developed higher median anti-Spike IgG levels compared to the other categories (1500 U/ml, 15–7,500 versus 780 U/ml, 0.8–12,500, *p* = 0.03). As expected, previous COVID-19 infection was associated with a higher frequency of seroconversion (*p* = 0.02) and higher median anti-Spike titers (5,850 U/ml, 24–12,500 versus 1,753 U/ml, 0.8–12,500, *p* < 0.001). Twenty-eight out of 393 patients (7%) had positive anti-Nucleocapsid antibodies, half of them reported a previous COVID-19 infections, and almost all seroconverted for anti-Spike. Finally, gender, inclusion criteria, and number of previous therapies did not seem to affect response to vaccine.

### Development of an Algorithm to Predict Seroconversion

In order to assess predictors of seroconversion, we performed multivariate logistic regression analysis: a diagnosis other than indolent lymphoma or CLL [odds ratio (OR) 8.5 (95% confidence interval, CI 4.1–17.6), *p* < 0.0001], lack of B-cell-depleting therapy (either small molecules or MoAbs) [OR 3.15 (1.7–5.9), *p* < 0.0001], and the presence of IgG levels higher than the lower limit of normality (700 mg/dl) [OR 2.2 (1.2-4.2), *p* = 0.01] were the only associated variables [AUC = 0.82 (95% CI 0.77–0.87)]. Based on logistic regression results, we calculated the predicted probability of vaccine response and developed a simple algorithm according to these 3 risk factors [(A) diagnosis of indolent lymphoma/CLL, (B) B-cell-depleting treatment, and (C) low IgG, **Table 2**]. The lowest response (11%) was predicted by the presence of all three risk factors, a low/intermediate one by risk factors A+B (22%) or A+C (29%), and an intermediate response by A alone (48%) or B+C (52%). Good responses were anticipated by the presence of risk factor B or C alone (71% and 77%, respectively) and the absence of any risk factor (89%). There was fair agreement between predicted and observed proportions (*p* = 0.57 from Hosmer–Lemeshow goodness-of-fit test).

**Table 2:** Predicted and observed proportions of SARS-CoV-2 anti-Spike seroconversion according to hematologic diagnosis, therapy, and immunoglobulin G (IgG) levels.

No. of patients	ADiagnosis of indolent lymphoma/CLL	BB-cell-depleting therapy	CIgG <700 mg/dl	Predicted probability	Observedproportion
24	Yes	Yes	Yes	0.11	0.17
20	Yes	Yes	No	0.22	0.15
7	Yes	No	Yes	0.29	0.29
10	Yes	No	No	0.48	0.50
31	No	Yes	Yes	0.52	0.58
22	No	Yes	No	0.71	0.64
44	No	No	Yes	0.77	0.70
121	No	No	No	0.89	0.91

CLL, chronic lymphocytic leukemia.

### Persistence of Anti-Spike Antibodies and Breakthrough SARS-CoV-2 Infections

**Table 3** shows the clinical features of patients re-tested for anti-Spike antibodies at 3 months from the last SARS-CoV-2 vaccine dose (*N* = 140, 36%). Overall, all patients who were positive at initial evaluation maintained anti-Spike IgG positivity at 3 months, although with a 37% decline of median titers (488 U/ml at 3 months versus 786 U/ml at first test). By comparing anti-Spike IgG titers at 3 months versus baseline, 2 patients displayed the same anti-Spike IgG titer, while 91 (65%) showed declining titers and 47 (34%) showed increasing ones. Patients with increasing titers had significantly higher neutrophils at baseline as compared with those with declining anti-Spike antibodies [3.5 × 10^9^/L (0.3–26) versus 2.7 × 10^9^/L (0.1–17), *p* = 0.01]. Interestingly, 2/5 patients on TKI for indolent lymphoma, and 3/3 patients on treatment with venetoclax plus hypomethylating agents increased their anti-Spike titer from baseline (median delta 273 U/ml, range 113–825). By multivariable regression analysis, no significant associations were noted among anti-Spike titer trends (increasing/decreasing) and disease types, inclusion criteria for vaccination, disease status, ongoing therapy, and recent transplant. Only patients with previous COVID-19 infection had a significantly higher increase of anti-Spike Ig titers from baseline as compared to the others [median delta 7,455 (23–12,500) U/ml versus 666 (0.8–12,500), *p* = 0.001].

**Table 3:** Clinical features of patients re-tested for anti-Spike antibodies at 3 months from the last SARS-CoV-2 vaccine dose, altogether and divided according to the trend of titers from baseline.

	Total (*n* = 138)	Declining (*n* = 91)	Growing (*n* = 47)
**Disease type**			
- AL	22	12	10
- Lymphoma	18	12	6
- CLL	3	1	2
- MDS	8	4	4
- MPN	45	30	15
- PCD	39	29	10
- Benign conditions	3	3	0
**Inclusion criteria**			
- Ongoing therapy	106	71	35
- < 6 months from end of therapy	8	6	2
- < 12 months from SCT	10	6	4
- Off therapy	11	6	5
**Ongoing therapy**			
- Small molecules for NHL	5	3	2
- MoAb	4	3	1
- MoAb + chemotherapy	3	2	1
- IMIDs and proteasome inhibitors	33	26	7
- HMA	9	2	7
- HMA + Venetoclax	3	0	3
- Chemotherapy	14	9	5
- Ruxolitinib	13	7	6
- Other TKI for MPN	28	21	7
- Immunosuppressants	3	3	0
- Complement inhibitors	2	2	0
**Disease status**			
- Response off therapy	17	12	5
- Response on therapy	100	67	33
- No response on therapy	11	5	6
- Active disease	7	5	2
**Transplant status**			
- No transplant	101	64	37
- Autologous SCT	26	19	7
- Allogenic SCT	11	8	3
**Previous COVID infection**			
- No	128	84	44
- Yes	10	7	3

AL, acute leukemias; CLL, chronic lymphocytic leukemia; IMIDs, immunomodulating agents; MDS, myelodysplastic syndrome; MoAb, monoclonal antibodies; MPN, myeloproliferative neoplasms; PCD, plasma cell dyscrasias; TKI, tyrosine kinase inhibitors.

Values are shown as total number.

Two patients displayed the same anti-Spike titer at re-testing.

Finally, 3 patients (2 acute leukemias, one subjected to SCT and one to recent chemotherapy, and 1 diffuse large B-cell lymphoma recently treated with chemotherapy and rituximab) who were previously negative for anti-Nucleocapsid antibodies seroconverted, indicating a likely breakthrough COVID-19 infection although asymptomatic in all of them.

## Discussion

Our results show that the rate of seroconversion to mRNA SARS-CoV-2 vaccines (72%) is lower in patients with hematologic diseases as compared to the healthy population (1–3). This response also appears to be clearly lower than that observed at our center in 3,475 healthcare workers, where only 0.2% had undetectable anti-Spike (12). This finding is in keeping with other reports (1–3) and underlines the importance of maintaining high levels of protective measures in this fragile population. Importantly, disease status and previous therapy lines, including SCT, should not discourage vaccination, since they did not affect seroconversion. Our analysis clearly showed that patients with lymphomas (particularly those with indolent NHL) and CLL have an intrinsic lower response to mRNA vaccine (50% unresponsive) that increases to 70% if another risk factor (B-cell-depleting treatment or hypogammaglobulinemia) is present, and to 89% if both coexist. While disease is a non-modifiable risk factor, Ig levels and treatment may vary overtime and may thus be considered for timing of vaccine administration. In particular, it may be considered to allow enough time (>6 months) from the last MoAb infusion and to hold on with small molecules if clinically feasible (i.e., subjects in long-term remission). This is also valid for multiple myeloma and myeloid malignancies, which generally responded better to m-RNA SARS-CoV-2 vaccine but showed lower rates of seroconversion in the case of MoAb therapy for multiple myeloma, venetoclax plus HMA for AML, and ruxolitinib for MPN. Furthermore, individual immunologic status should be assessed (lymphocyte and neutrophil values, IgG, IgA, and IgM levels), and it may be considered that patients should wait for immune reconstitution, particularly IgG recovery, before vaccination. As regards the persistence of anti-Spike titers, it was 100% in the subgroup of patients re-tested at 3 months from the last vaccine dose, irrespective of disease type, progression status, and treatment. Our findings differ from those by Tadmor et al. who described anti-Spike persistence in 73% of CLL patients only (13). Intriguingly, in our study, anti-Spike titer increased from baseline in more than one-third of subjects, particularly in those with previous COVID-19 infection and with higher neutrophils, possibly in keeping with a more preserved hematopoiesis. Increasing titers were noted even in 50% of patients receiving small molecules for indolent lymphomas and in all AML patients on venetoclax, indicating that once obtained, seroconversion persists even in this at-risk population. Finally, the 3 patients who developed breakthrough anti-Nucleocapsid positivity, indicative of SARS-CoV-2 infection, were all asymptomatic, underlining the importance of vaccinating subjects with hematologic diseases.

Even if our study carries several limitations, particularly regarding the heterogeneity of the diseases included and related treatments, we believe that it provides a cross-section analysis of a large hematologic series vaccinated in a real-world setting. Future investigations may further validate and enrich our algorithm by optimizing vaccine strategies for COVID-19 as well as for other infectious agents.

In conclusion, our data show that about 70% of hematologic patients respond to SARS-CoV-2 mRNA vaccines with anti-Spike titer persistence in 100% of responders at 3 months. Along with non-modifiable risk factors for non-response (i.e., diagnosis of indolent lymphoma or CLL), the efficacy of SARS-CoV-2 vaccination may be influenced by recent/ongoing therapies and by the immunologic status of the patient as measured by Ig levels. This suggests allowing enough time from the last MoAb infusion, holding on with small molecules if clinically feasible, and waiting for IgG recovery before vaccination.

## Data Availability Statement

The original contributions presented in the study are included in the article/supplementary material. Further inquiries can be directed to the corresponding author.

## Ethics Statement

The study was approved by Comitato Etico Istituto Lazzaro Spallanzani, Rome, code HECOVID. The patients/participants provided their written informed consent to participate in this study.

## Author Contributions

BF, JG, RC, and LB designed the study and wrote the experimental protocol. BF, MB, WB, and LB wrote the manuscript. BF, MB, NR, FCa, JG, CB, RC, MV, GG, AN, LP, FR, MS, WB, and LB followed patients and collected data. DC performed statistical analysis. FCe supervised serologic tests. All authors contributed to the article and approved the submitted version.

## Conflict of Interest

The authors declare that the research was conducted in the absence of any commercial or financial relationships that could be construed as a potential conflict of interest.

## Publisher’s Note

All claims expressed in this article are solely those of the authors and do not necessarily represent those of their affiliated organizations, or those of the publisher, the editors and the reviewers. Any product that may be evaluated in this article, or claim that may be made by its manufacturer, is not guaranteed or endorsed by the publisher.

## References

[1] Greenberger LM Saltzman LA Senefeld JW Johnson PW DeGennaro LJ Nichols GL. Antibody Response to SARS-CoV-2 Vaccines in Patients With Hematologic Malignancies. Cancer Cell (2021) 39(8):1031–3. doi: 10.1016/j.ccell.2021.07.012 PMC8295014 34331856

[2] Maneikis K Šablauskas K Ringelevičiūtė U Vaitekėnaitė V Čekauskienė R Kryžauskaitė L. Immunogenicity of the BNT162b2 COVID-19 mRNA Vaccine and Early Clinical Outcomes in Patients With Haematological Malignancies in Lithuania: A National Prospective Cohort Study. Lancet Haematol (2021) 8(8):e583–92. doi: 10.1016/S2352-3026(21)00169-1 PMC8253543 34224668

[3] Malard F Gaugler B Gozlan J Bouquet L Fofana D Siblany L. Weak Immunogenicity of SARS-CoV-2 Vaccine in Patients With Hematologic Malignancies. Blood Cancer J (2021) 11(8):142. doi: 10.1038/s41408-021-00534-z 34376633 PMC8353615

[4] Perry C Luttwak E Balaban R Shefer G Morales MM Aharon A. Efficacy of the BNT162b2 mRNA COVID-19 Vaccine in Patients With B-Cell non-Hodgkin Lymphoma. Blood Adv (2021) 5(16):3053–61. doi: 10.1182/bloodadvances.2021005094 PMC8362658 34387648

[5] Ehmsen S Asmussen A Jeppesen SS Nilsson AC Østerlev S Vestergaard H. Antibody and T Cell Immune Responses Following mRNA COVID-19 Vaccination in Patients With Cancer. Cancer Cell (2021) 39(8):1034–6. doi: 10.1016/j.ccell.2021.07.016 PMC8313483 34348121

[6] Griffiths EA Segal BH. Immune Responses to COVID-19 Vaccines in Patients With Cancer: Promising Results and a Note of Caution. Cancer Cell (2021) 39(8):1045–7. doi: 10.1016/j.ccell.2021.07.001 PMC8253695 34242573

[7] Available at: https://www.gazzettaufficiale.it/eli/gu/2021/03/24/72/sg/pdf (Accessed 4th, September 2021).

[8] Kohmer N Westhaus S Rühl C Ciesek S Rabenau HF. Brief Clinical Evaluation of Six High-Throughput SARS-CoV-2 IgG Antibody Assays. J Clin Virol (2020) 129:104480. doi: 10.1016/j.jcv.2020.104480 32505777 PMC7263247

[9] Müller L Ostermann PN Walker A Wienemann T Mertens A Adams O. Sensitivity of Anti-SARS-CoV-2 Serological Assays in a High-Prevalence Setting. Eur J Clin Microbiol Infect Dis (2021) 40:1063–71. doi: 10.1007/s10096-021-04169-7 PMC7856849 33534090

[10] World Health Organization. Establishment of the WHO International Standard and Reference Panel for Anti-SARSCoV-2 Antibody. WHO/Bs/2020.2403 (2020). Available at: https://www.who.int/publications/m/item/WHO-BS-2020.240.

[11] First WHO International Standard Anti-SARS-CoV-2 Immunoglobulin (Human). Available at: https://www.nibsc.org/products/brm_product_catalogue/detail_page.aspx?catid=20/136. 10.1016/S0140-6736(21)00527-4 PMC7987302 33770519

[12] Lombardi A Consonni D Oggioni M Bono P Uceda Renteria S Piatti A. SARS-CoV-2 Anti-Spike Antibody Titres After Vaccination With BNT162b2 in Naïve and Previously Infected Individuals. J Infect Public Health (2021) 14(8):1120–2. doi: 10.1016/j.jiph.2021.07.005 PMC8285930 34293641

[13] Tadmor T Benjamini O Braester A Rahav G Rokach L. Antibody Persistence 100 Days Following the Second Dose of BNT162b mRNA Covid19 Vaccine in Patients With Chronic Lymphocytic Leukemia. Leukemia (2021) 35(9):2727–30. doi: 10.1038/s41375-021-01380-5 PMC8353933 34376803

